# Roles of the ubiquitin ligase CUL4B and ADP-ribosyltransferase TiPARP in TCDD-induced nuclear export and proteasomal degradation of the transcription factor AHR

**DOI:** 10.1016/j.jbc.2021.100886

**Published:** 2021-06-16

**Authors:** Mercedes Paz Rijo, Silvia Diani-Moore, Chenyi Yang, Pengbo Zhou, Arleen B. Rifkind

**Affiliations:** 1 Department of Pharmacology, Weill Cornell Medicine, New York, New York, USA; 2 Department of Pathology and Laboratory Medicine, Weill Cornell Medicine, New York, New York, USA

**Keywords:** 2,3,7,8-tetrachlorodibenzo-*p*-dioxin (TCDD, dioxin), aryl hydrocarbon receptor (AHR), cullin 4B (CUL4B), cytochrome P450 (CYP450), E3 ubiquitin ligase, interferon beta 1 (Ifnb1), mouse embryonic fibroblasts (MEFs), nuclear export, protein proteasomal degradation, TCDD-inducible poly(ADP-ribose) polymerase (TiPARP, PARP7, ARTD14), +siTiPARP, silencing TiPARP by siRNA, 3pRNA, 5'-triphosphate RNA, AHR, aryl hydrocarbon receptor, ARNT, aryl hydrocarbon receptor nuclear translocator, CUL4B, cullin 4B, FBS, fetal bovine serum, Infb1, interferon beta 1, LMB, leptomycin B, MEF, mouse embryonic fibroblast, MEF^*Cul4b*-null^, MEF cell line in which the *Cullin 4B* gene had been knocked out, NES, nuclear export signal, RT-qPCR, real-time quantitative PCR, sgRNA, single-guide RNA, TCDD, 2,3,7,8-tetrachlorodibenzo-*p*-dioxin, TiPARP, TCDD-inducible poly(ADP-ribose) polymerase

## Abstract

The aryl hydrocarbon receptor (AHR) is a transcription factor activated by exogenous halogenated polycyclic aromatic hydrocarbon compounds, including the environmental toxin TCDD, 2,3,7,8-tetrachlorodibenzo-*p*-dioxin, and naturally occurring dietary and endogenous compounds. The activated AHR enhances transcription of specific genes including phase I and phase II metabolism enzymes and other targets genes such as the TCDD-inducible poly(ADP-ribose) polymerase (TiPARP). The regulation of AHR activation is a dynamic process: immediately after transcriptional activation of the AHR by TCDD, the AHR is exported from the nucleus to the cytoplasm where it is subjected to proteasomal degradation. However, the mechanisms regulating AHR degradation are not well understood. Here, we studied the role of two enzymes reported to enhance AHR breakdown: the cullin 4B (CUL4B)^AHR^ complex, an E3 ubiquitin ligase that targets the AHR and other proteins for ubiquitination, and TiPARP, which targets proteins for ADP-ribosylation, a posttranslational modification that can increase susceptibility to degradation. Using a WT mouse embryonic fibroblast (MEF) cell line and an MEF cell line in which CUL4B has been deleted (MEF^*Cul4b*-null^), we discovered that loss of CUL4B partially prevented AHR degradation after TCDD exposure, while knocking down TiPARP in MEF^*Cul4b*-null^ cells completely abolished AHR degradation upon TCDD treatment. Increased TCDD-activated AHR protein levels in MEF^*Cul4b*-null^ and MEF^*Cul4b*-null^ cells in which TiPARP was knocked down led to enhanced AHR transcriptional activity, indicating that CUL4B and TiPARP restrain AHR action. This study reveals a novel function of TiPARP in controlling TCDD-activated AHR nuclear export and subsequent proteasomal degradation.

2,3,7,8-Tetrachlorodibenzo-*p*-dioxin (TCDD, dioxin), a byproduct of incineration and other industrial processes, is a ubiquitous environmental contaminant whose toxic effects, widely observed in different species, include developmental defects, cancer, a wasting syndrome, hepatosteatosis, thymus involution, and dysregulation of immune responses (1, 2, 3, 4). TCDD is the best-known ligand of the aryl hydrocarbon receptor (AHR) (5), but other halogenated and polycyclic aromatic hydrocarbon compounds as well as naturally occurring dietary and endogenous compounds, such as tryptophan and indole metabolites, have also been identified as AHR ligands (6, 7).

AHR transcriptional activation is a dynamic process: an inactive AHR that is not bound to a ligand (either endogenous or exogenous) resides in the cytoplasm in a complex with several different proteins (*i.e.*, heat shock protein 90, AHR-interacting protein, and AHR-activated 9 protein (8)). Upon ligand binding, the AHR moves into the nucleus where it dimerizes with the aryl hydrocarbon receptor nuclear translocator (ARNT) protein to produce an AHR/ARNT heterodimer that activates gene transcription by binding to dioxin-responsive elements, specific DNA sequences in the promoter regions of AHR-responsive genes, that is, phase I (oxidation) and phase II (conjugation) drug-metabolism enzymes (including cytochrome P450 enzymes in the CYP1A and CYP1B families (mammalian *cyp1a1*, *cyp1a2*, and *cyp1b1*), and many other targets including TCDD-inducible poly(ADP-ribose) polymerase (TiPARP) (also known as PARP7 and ARTD14)), and the aryl hydrocarbon receptor repressor (8, 9).

Simultaneously with activation of AHR-mediated gene transcription, the AHR begins to be degraded and AHR levels decline (10). Proteolytic degradation of transcription factors is a known mechanism for regulating signaling pathways (11), and although the phenomenon of AHR degradation has been known for many years, the process by which it occurs is not well understood. Through the late 1990s and early 2000s, a series of articles by Richard Pollenz *et al.* made inroads into the understanding of the mechanism, revealing that, after AHR ligand activation and translocation to the nucleus, a nuclear export signal (NES) on the N-terminal region of the AHR promotes its movement from the nucleus to the cytoplasm where the AHR is degraded *via* the 26S proteasome machinery (10, 12, 13). Inhibition of the ubiquitin–proteasome pathway was shown to increase the levels of the AHR–ARNT complex in the nucleus leading to ‘superinduction’ of TCDD-induced *Cyp1a1* mRNA levels (14), thus indicating that AHR degradation is a mechanism for restraining the transcriptional activity of the AHR.

E3 ubiquitin ligase complexes catalyze ubiquitination of specific proteins, targeting them for proteasomal degradation and are involved in many cellular functions and biological processes (15). In 2007, Ohtake *et al.* (16) identified the ligand-activated AHR as an E3 ubiquitin ligase, part of an atypical cullin–RING ligase 4B complex (named CUL4B^AHR^), where CUL4B functions as a scaffold mediating interaction among the different components and AHR as the substrate-specific adaptor providing specificity for targeting proteins for ubiquitination (16, 17, 18). CUL4B interacts with the AHR *via* its N-terminal extension (16). Cullin 4A, which shares a mostly identical amino acid sequence with CUL4B but lacks the N-terminal extension (15), does not interact with the AHR (16). Other targets of the CUL4B^AHR^ complex include the estrogen receptor (16), androgen receptor (AR) (16), β-catenin (17), peroxisome proliferator-activated receptor γ (18), and the AHR (16). This mechanism could contribute to regulate activated AHR levels. More recently, the AHR target gene TiPARP, a mono-ADP-ribosyltransferase, was shown to affect stability of the activated AHR, thus knocking out TiPARP increased AHR protein levels in both a mouse embryonic fibroblast (MEF) cell line and liver, an effect accompanied by enhanced AHR transcriptional activity by TCDD (19, 20) and supporting a role for TiPARP in regulating AHR levels and activity.

In the studies reported here, we sought to investigate the effects of loss of CUL4B and TiPARP on activated AHR degradation, cellular localization, and activity to better understand how these two factors regulate the AHR after its ligand activation. To this end, we used TCDD as a prototype ligand to activate the AHR, immortalized MEF cell line in which the *Cullin 4B* gene had been knocked out (MEF^*Cul4b*^^-null^), and CRISPR/Cas9 technology to knock down TiPARP.

## Results

### Loss of CUL4B increases AHR protein levels and TCDD-activated AHR transcriptional activity

**Figure 1:**
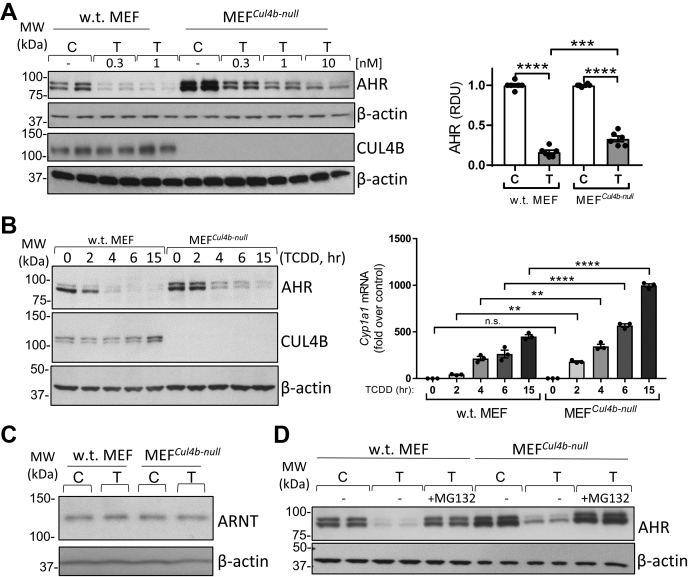
**Loss of CUL4B increases AHR protein levels and AHR transcriptional activity in MEF cells.***A*, WT and MEF^*Cul4b-null*^ cells were treated with solvent dioxane or 0.3, 1, and 10 nM TCDD for 6 h. *Left panels*, cell homogenates were used for Western blotting (WB) analysis with antibodies against AHR, CUL4B, and β-actin; *right bar graph* shows densitometry analysis of AHR protein levels from n = 5 experiments using TCDD (1 nM) with 1 or 2 replicates per treatment group for each experiment (*p* ≤ 0.0001, T *versus* C for both WT MEF and MEF^*Cul4b*-null^; *p* = 0.0004, T (WT MEF) *versus* T MEF^*Cul4b*-null^). *B*, WT MEF and MEF^*Cul4b-null*^ cells treated with TCDD (0.3 nM) for 0, 2, 4, 6, and 15 h were used to prepare homogenates for WB analysis with antibodies indicated in the figure (*left panels*) or to extract RNA and analyze *Cyp1a1* mRNA levels (an index of AHR transcriptional activity) by RT-qPCR (*right bar graph*); WT *versus* MEF^*Cul4b*-null^: *p* = 0.0024 (T 2 h), *p* = 0.0005 (T 4 h), *p* < 0.0001 (T 6 h), *p* < 0.0001 (T 15 h). *C*, WB using homogenates of WT MEF and MEF^*Cul4b-null*^ cells (TCDD, 10 nM) with antibodies against ARNT and β-actin. Results are representative of n = 2 independent experiments. *D*, representative results for WB using homogenates of WT MEF and MEF^*Cul4b-null*^ cells treated with TCDD (1 nM, 6 h) ± MG132 (50 μM). For bar graphs in panels *A* and *B*, one-way ANOVA was used to calculate differences among the means and Tukey's honestly significant difference (HSD) test was used as a post hoc test. For this and other figures, bar graphs represent means ± SE with experimental replicates shown as *black-filled circles*; *∗p* ≤ 0.05; *∗∗p* ≤ 0.01; ∗∗∗*p* ≤ 0.001; ∗∗∗∗*p* ≤ 0.0001. AHR, aryl hydrocarbon receptor; ARNT, aryl hydrocarbon receptor nuclear translocator; C, control (solvent); CUL4B, cullin 4B; MEF, mouse embryonic fibroblast; MEF^*Cul4b*-null^, MEF cell line in which CUL4B has been knocked out; n.s., not significant; RDU, relative densitometry units; RT-qPCR, real-time quantitative PCR; T, TCDD; T, 2,3,7,8-tetrachlorodibenzo-*p*-dioxin.

To study the contribution of the CUL4B^AHR^ E3 ligase complex to AHR degradation by TCDD, we used an MEF^*Cul4b*-null^. Immortalized MEF^*Cul4b*-null^ cell line was derived from a CUL4B KO mouse described in ref (21). We treated WT and MEF^*Cul4b*-null^ cells with several doses of TCDD (0.3, 1, and 10 nM) or solvent (control) for 6 h. The mean decrease of AHR levels by TCDD at 1 nM (n = 5 independent experiments with 1 or 2 replicates per treatment group) was 84% in WT cells and 65% in MEF^*Cul4b*-null^ cells (Fig. 1*A*), indicating that loss of CUL4B diminished but did not prevent AHR degradation. Figure 1*A* also shows that CUL4B is detected and not affected by TCDD in the WT MEF cell line while CUL4B protein is absent in MEF^*Cul4b*-null^ cell line. To study the changes in AHR abundance after its activation by TCDD in correlation with AHR transcriptional activity, we conducted a time course experiment in WT MEF and MEF^*Cul4b*-null^ cells treated with TCDD for 2, 4, 6, and 15 h and we measured both AHR levels and *Cyp1a1* mRNA levels, a hallmark of TCDD–AHR activation (22, 23). Left panels in Figure 1*B* show that after TCDD (T) treatment, levels of the AHR decreased with time in WT MEF cells by −43% (T, 2 h), −77% (T, 4 h), −89% (T, 6 h), and −91% (T, 15 h), consistent with AHR degradation occurring once the AHR is activated by TCDD. In MEF^*Cul4b*-null^ cells, AHR protein diminished less after TCDD treatment with time, −7%, (T 2 h), −64% (T 4 h), −68% (T 6 h), and −77% (T 15 h), suggesting that there is less AHR degradation with the loss of CUL4B (for each cell line, percentages were calculated comparing relative densitometry units of AHR levels at each time point with AHR levels before TCDD treatment). Figure 1*B*, right bar graph, shows that *Cyp1a1* mRNA levels were increased by TCDD also in a time-dependent manner and were higher at each time point in MEF^*Cul4b*-null^ cells than WT MEF cells: MEF^*Cul4b*-null^ *versus* WT cells, +4.3-fold (T 2 h), +61% (T 4 h), +2.16-fold (T 6 h), +2.21-fold (T 15 h). These data show that diminished AHR degradation with loss of CUL4B leads to increased AHR transcriptional activity. Figure 1*C* shows that the AHR dimerization partner, ARNT, was not decreased by TCDD treatment in agreement with a previous report by Song and Pollenz (24), nor was ARNT affected by CUL4B KO.

We next asked whether the decrease in AHR protein levels observed in MEF^*Cul4b*-null^ cells by TCDD was attributable to proteasomal degradation. Figure 1*D* shows that cotreatment with TCDD and the proteasome inhibitor MG132 not only prevented the decrease of AHR levels by TCDD in WT cells but also prevented AHR degradation in MEF^*Cul4b*-null^ cells, indicating that the 26S proteasome is responsible for the decrease of AHR protein levels by TCDD also in the absence of the CUL4B^AHR^ ubiquitin ligase.

### TiPARP promotes AHR protein degradation in the absence of CUL4B. Loss of both TiPARP and CUL4B completely prevented TCDD-induced AHR degradation

**Figure 2:**
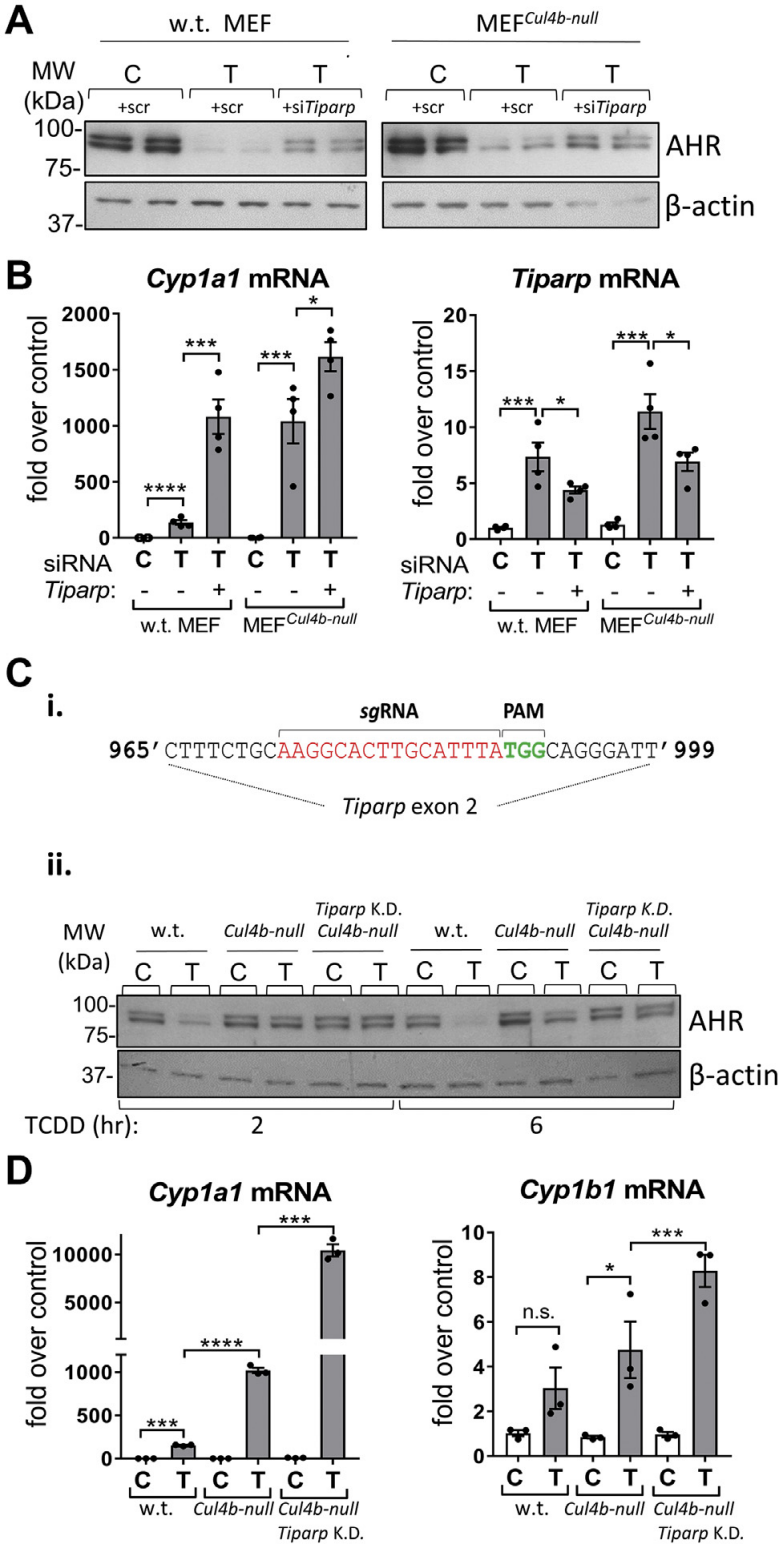
**TiPARP leads to TCDD-activated AHR degradation in the absence of CUL4B.***A*, WB using homogenates of WT MEF and MEF^*Cul4b-null*^ cells transfected with dsRNAs targeting mouse TiPARP (+siTiPARP) or nontargeted control dsRNAs (+scr) and treated with solvent or TCDD (10 nM) for 6 h; antibodies against AHR and β-actin were used. *B*, RT-qPCR for *Cyp1a1* and *Tiparp* using RNA extracted from WT MEF and MEF^*Cul4b-null*^ cells treated as in panel *A* (n = 3 independent experiments). *C*, i, scheme showing the sequence in *Tiparp* exon 2 targeted by single-guide RNA (*sg*RNA) used for the CRISPR/Cas9-mediated deletion of *Tiparp* in MEF^*Cul4b-null*^ cells to abolish TiPARP. ii, *panels*, WB using homogenates of WT MEF, MEF^*Cul4b-null*^, and MEF^*Cul4b*-null*/Tiparp*^^K.D.^ cells treated with solvent or TCDD (1 nM, 6 h) and antibodies for AHR and β-actin. *D*, RT-qPCR for *Cyp1a1* and *Cyp1b1* using RNA extracted from WT MEF, MEF^*Cul4b-null*^, and MEF^*Cul4b-null*^ with TiPARP knocked down cells treated with TCDD (1 nM, 6 h) (n = 3). For bar graphs in panels *B* and *D*, one-way ANOVA was used to calculate differences among the means and Tukey's honestly significant difference (HSD) test was used as a post hoc test. +scr, scrambled siRNA; +siTiPARP, silencing TiPARP by siRNA; AHR, aryl hydrocarbon receptor; *C*, control (solvent); CUL4B, cullin 4B; MEF, mouse embryonic fibroblast; MEF^*Cul4b*-null^, MEF cell line in which the *Cullin 4B* gene had been knocked out; n.s., not significant; PAM, protospacer adjacent motif; RDU, relative densitometry units; RT-qPCR, real-time quantitative PCR; T, TCDD, T, 2,3,7,8-tetrachlorodibenzo-p-dioxin; TiPARP, TCDD-inducible poly(ADP-ribose) polymerase; WB, Western blotting.

The AHR target gene TiPARP has been reported to promote AHR degradation *via* its ADP-ribosylation activity (20), and PARP-mediated ADP-ribosylation has been shown to signal for ubiquitination and proteasomal degradation of proteins (25). Thus, we asked whether TiPARP can lead to AHR degradation after TCDD treatment in the absence of CUL4B or whether CUL4B is required for TiPARP action. Figure 2*A* shows that silencing TiPARP by siRNA (+siTiPARP) increased AHR protein levels in both TCDD treated WT MEF and MEF^*Cul4b*-null^ cells compared with cells treated with TCDD + scrambled siRNA, indicating that TiPARP can promote degradation of the AHR even in the absence of CUL4B. The combination of TiPARP knockdown and loss of CUL4B in MEF^*Cul4b*-null^ produced higher AHR protein levels than TiPARP knockdown or loss of CUL4B alone, indicating that TiPARP and CUL4B have combined effects in promoting AHR degradation. Higher AHR protein levels after silencing TiPARP in TCDD-treated WT MEF and MEF^*Cul4b*-null^ cells were accompanied by increased transcriptional activity of the AHR, as shown by increased mRNA levels of the AhR target gene *Cyp1a1* (Fig. 2*B*, left bar graph).

+siTiPARP achieved only about a 40% decrease in *Tiparp* mRNA levels (Fig. 2*B*, right bar graph), thus the residual TiPARP could still account for the lower AHR protein levels in TCDD + siTiPARP–treated MEF^*Cul4b*-null^ cells than AHR protein levels in control (scrambled siRNA) MEF^*Cul4b*-null^ cells (Fig. 2*A*, upper right panel). To eliminate TiPARP more completely, we used CRISPR/Cas9 technology with a single-guide RNA (sgRNA) targeting exon 2 of the TiPARP gene (Fig. 2*C*i) in MEF^*Cul4b-null*^ cells as described in Experimental procedures. To assess the loss of TiPARP, we performed Western blot analysis using several anti-TiPARP antibodies (including several commercially available and a custom-made antibody that successfully recognized chicken TiPARP (26)), but their lack of specificity did not allow the validation of the loss of TiPARP protein by this method. Therefore, we assessed the presence of mutations in the TiPARP gene sequence by ultra-deep sequencing of PCR amplicons of the TiPARP gene region containing exon 2. The sequence analysis revealed that we obtained a 100% homozygous mutant cell line that harbors 97% of frameshift mutations leading to a premature stop codon within the ORF of the TiPARP gene (Fig. S1). WT MEF, MEF^*Cul4b*-null^ cells, and MEF^*Cul4b*-null^ cells in which TiPARP was knocked down (MEF*^Cul4b^*^-null/^*^Tiparp^* ^K.D.^) were treated with TCDD (1 nM) or solvent for 2 or 6 h and analyzed by Western blotting to assess TCDD effects on AHR protein levels in these three cell lines. Figure 2*C*ii shows that after 2-h of treatment, TCDD decreased AHR protein levels by 70% in the WT MEF cells, while decreasing the AHR by 20% in the MEF^*Cul4b*-null^ cells; in MEF^*Cul4b*-null/*Tiparp*^ ^K.D.^ cells, TCDD did not decrease AHR protein levels. After 6 h of treatment, the AHR protein was barely detected in TCDD-treated WT MEF cells and was decreased by about 55% in TCDD-treated MEF^*Cul4b*-null^ cells. Strikingly, in MEF^*Cul4b*-null/*Tiparp*^ ^K.D.^ cells, AhR degradation by TCDD was completely prevented. Higher AHR protein levels correlated with higher AHR transcriptional activity as *Cyp1a1* and *Cyp1b1* mRNAs were higher in MEF^*Cul4b*-null/*Tiparp*^ ^K.D.^ cells than MEF^*Cul4b*-null^ cells after TCDD treatment (Fig. 2*D*). Together, these results indicate that AHR degradation by TCDD is completely abolished in the absence of both CUL4B and TiPARP and that TiPARP promotes AHR degradation in the absence of CUL4B.

### Loss of TiPARP and CUL4B affects the induction of *Infb1* mRNA by a viral motif

**Figure 3:**
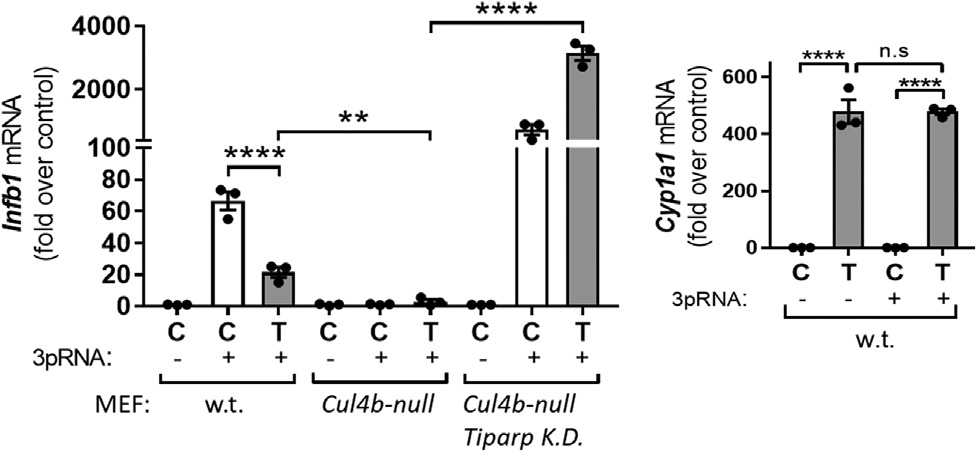
**Effects of loss of CUL4B and TiPARP on viral induced *Infb1* mRNA.***Left bar graph*, RT-qPCR of *Infb1* mRNA using RNA extracted from WT MEF, MEF^*Cul4b*-null^, and MEF^*Cul4b*-null*/Tiparp*^^K.D.^ transfected with 5'-triphosphate RNA (+3pRNA) or control RNA (−3pRNA) and treated with solvent or TCDD (1 nM, 24 h). *Right bar graph*, RT-qPCR analysis of *Cyp1a1* mRNA in WT MEF cells transfected with ±3pRNA and treated with the solvent or TCDD (1 nM, 24 h). Bar graphs represent the means ± SEM (n = 3). One-way ANOVA was used to calculate differences among the means, and Tukey's honestly significant difference (HSD) test was used as a post hoc test. C, control (solvent); CUL4B, cullin 4B; Infb1, interferon beta 1; MEF, mouse embryonic fibroblast; MEF^*Cul4b*-null^, MEF cell line in which the *Cullin 4B* gene had been knocked out; n.s., not significant; RDU, relative densitometry units; RT-qPCR, real-time quantitative PCR; T, TCDD, T, 2,3,7,8-tetrachlorodibenzo-p-dioxin; TiPARP, TCDD-inducible poly(ADP-ribose) polymerase.

We next asked whether the increased AHR protein levels with the loss of CUL4B and TiPARP could have biological consequences other than enhancing induction of AHR target genes. The induction of the cytokine interferon beta 1 (Infb1) is part of the first line of defense against viral infection in the host cell (27). It has been recently reported that AHR activation curtails virus-induced Infb1 mRNA, an effect mediated by TiPARP induction in the host cell (28). Thus, TiPARP was shown to ADP-ribosylate and diminish the activity of the TANK-binding kinase 1, which is a stimulator of *Infb1* mRNA induction (28). Thus, we examined the effects of TCDD on the virus-induced *Infb1* mRNA in WT MEF, MEF^*Cul4b*-null^, and MEF^*Cul4b*-null/*Tiparp*^ ^K.D.^ cells. We transfected the three cell lines with 5'-triphosphate RNA (3pRNA, a viral motif that leads to the induction of Infb1 mRNA) followed by treatment of the cells with TCDD or solvent for 6 h. Figure 3 (left bar graph) shows that in WT cells, 3pRNA increased the induction of Infb1 mRNA levels (control + 3pRNA *versus* control - 3pRNA) and TCDD treatment decreased 3pRNA-induced Infb1 mRNA. Strikingly, 3pRNA did not induce Infb1 mRNA in both control and TCDD-treated MEF^*Cul4b*-null^ cells. Loss of both TiPARP and CUL4B (MEF^*Cul4b*-null/*Tiparp*^ ^K.D.^ cells) greatly increased the induction of Ifnb1 mRNA by 3pRNA in both control and TCDD-treated cells, consistent with a role for TiPARP in suppressing 3pRNA-induced Ifnb1 mRNA by TCDD-activated AHR. WT MEF cells transfected with 3pRNA had similar TCDD-induced Cyp1a1 mRNA compared with cells transfected with a control RNA motif (Fig. 3, right bar graph), indicating that 3pRNA did not alter TCDD-activated AHR transcriptional activity in these experimental conditions. The findings reveal that loss of CUL4B or TiPARP can have significant biological consequences as exemplified here by changes in virally induced Infb1 mRNA.

### Inhibition of AHR nuclear export leads to nuclear accumulation of the AHR in TCDD-treated WT cells

**Figure 4:**
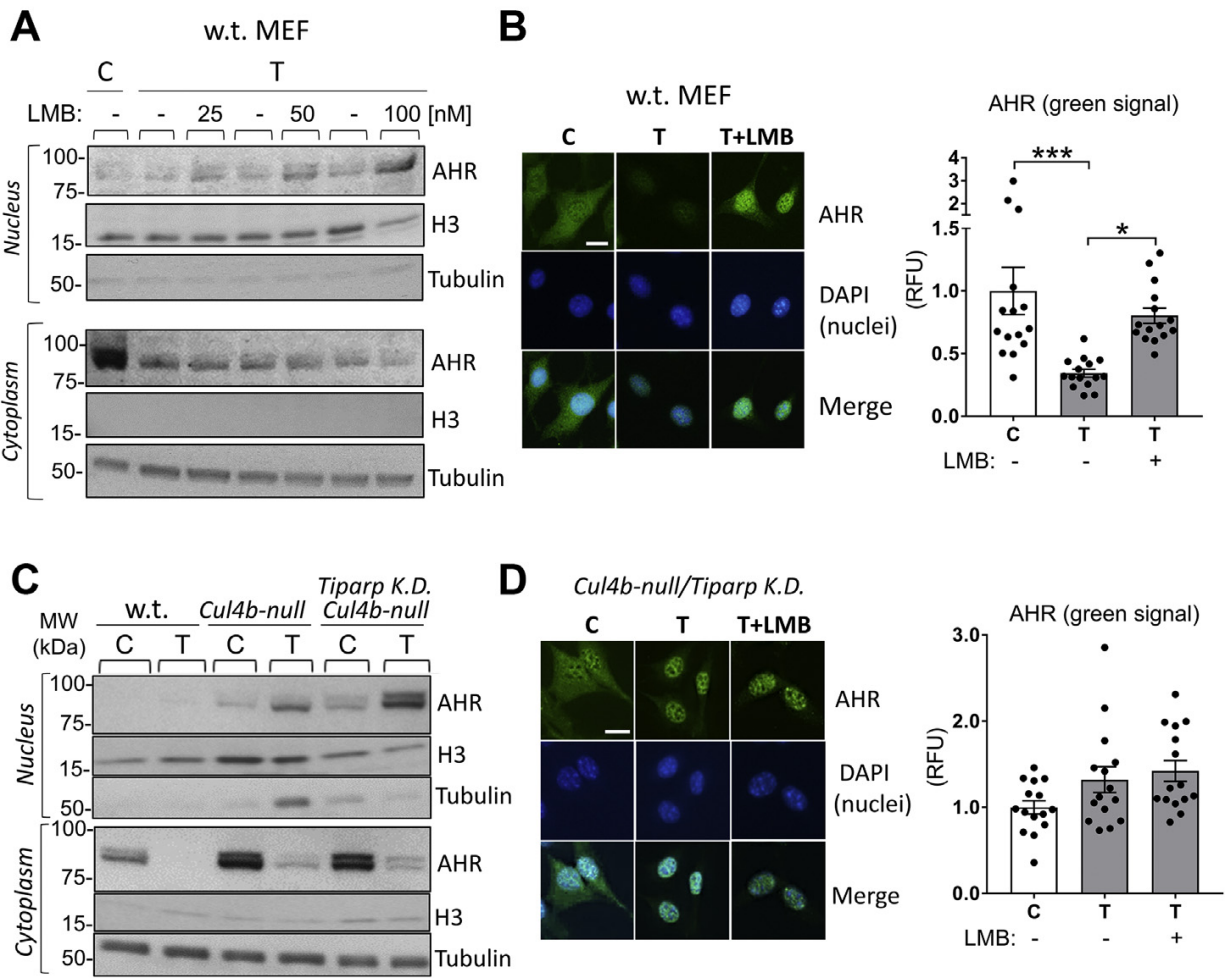
**Inhibition of the nuclear export of the TCDD-activated AHR by LMB leads to inhibition of AHR degradation and AHR nuclear accumulation; loss of CUL4B and TiPARP leads to TCDD-activated AHR accumulation in the nucleus.***A*, WB using nuclear- and cytoplasmic-enriched subcellular fractions of WT MEF cells treated with the solvent or TCDD (1 nM) with or without LMB (25, 50, and 100 nM) for 6 h. Antibodies against AHR, histone 3 (H3, nuclear marker), and α/β tubulin (cytoplasmic marker) were used. *B*, *left panels*, representative images of immunofluorescence analysis in WT MEF cells treated with the solvent or TCDD (1 nM, 6 h) with or without LMB (50 nM, overnight) using an AHR antibody (FITC, *green*). DAPI, nuclear marker (*blue*). Merge, visualization of both AHR and nuclear signals. *Right bar graph*, means ± SEM (n = 15 cells per treatment group) of AHR fluorescence signal (*green*). The *white* scale bar represents 20 μm. *C*, WB using nuclear- and cytoplasmic-enriched subcellular fractions of WT MEF, MEF^*Cul4b-null*^, and MEF^*Cul4b*-null*/Tiparp*^^K.D.^ cells treated with the solvent or TCDD (1 nM, 6 h). Antibodies against AHR, histone 3 (H3, nuclear marker), and α/β tubulin (cytoplasmic marker) were used. *D*, *left panels*, representative images of immunofluorescence analysis in MEF^*Cul4b*-null*/Tiparp*^^K.D.^ treated with the solvent or TCDD (1 nM, 6 h) with or without LMB (50 nM, overnight) using an AHR antibody (FITC, *green*). DAPI, nuclear marker (*blue*). Merge, visualization of both AHR and nuclear signals. *Right bar graph*, means ± SEM (n = 15 cells per treatment group) of AHR fluorescence signal (*green*). One-way ANOVA was used to calculate differences among the means, and Tukey's honestly significant difference (HSD) test was used as a post hoc test. The *white* scale bar represents 20 μm. AHR, aryl hydrocarbon receptor; C, control (solvent); CUL4B, cullin 4B; DAPI, 4′,6-diamidino-2-phenylindole; LMB, leptomycin B; MEF, mouse embryonic fibroblast; MEF^*Cul4b*-null^, MEF cell line in which the *Cullin 4B* gene had been knocked out; n.s., not significant; RDU, relative densitometry units; T, TCDD, T, 2,3,7,8-tetrachlorodibenzo-p-dioxin; WB, Western blotting.

Davarinos and Pollenz (12) showed, using HepG2 and Hepa-1 cell lines, that the TCDD-activated AHR requires export from the nucleus to the cytoplasm to be degraded by the 26S proteasome. We assessed the effect of inhibiting protein export from the nucleus on AHR stability using WT MEF cells. We treated the cells with solvent or TCDD and different concentrations of leptomycin B (LMB), an inhibitor of chromosome maintenance region 1 (also known as exportin 1) (29), which inhibits the nuclear export of proteins including the AHR (12). After 6-h treatment, LMB led to accumulation of the AHR in the nucleus in TCDD-treated WT MEF cells in a dose-dependent manner (Fig. 4*A*, upper panels). LMB decreased AHR protein levels in the cytoplasm, consistent with the inhibition of AHR nuclear export (Fig. 4*A*, lower panels). To validate these findings, we studied the subcellular localization of the AHR by immunofluorescence in WT MEF cells treated with the solvent or TCDD (1 nM, 6 h) with or without LMB (50 nM, a dose that did not alter cell morphology). Figure 4*B* shows that the AHR (green signal) resides largely in the cytoplasm of control cells and was decreased by TCDD by about 80% (bar graph). The remaining AHR protein localized in the nucleus, as shown by the overlapping of the AHR signal and the nuclear stain 4′,6-diamidino-2-phenylindole (blue). Cotreatment with TCDD + LMB increased the AHR signal in the nucleus 2.4-fold, consistent with the results shown in Figure 4*A* and by Davarinos and Pollenz (12) who reported that inhibiting nuclear export leads to accumulation of the ligand-activated AHR in the nucleus.

### Loss of CUL4B and TiPARP increases nuclear AHR levels

To study the roles of CUL4B and TiPARP in AHR nuclear localization, we treated MEF^*Cul4b*-null^ and MEF^*Cul4b*-null/*Tiparp*^ ^K.D.^ cells along with WT MEF cells with solvent (control) or TCDD (1 nM) for 6 h and prepared nuclear and cytoplasmic fractions. Figure 4*C* shows that AHR protein levels were increased by about 2.7-fold in the nuclear fraction of TCDD-treated MEF^*Cul4b*-null^ cells compared with the control MEF^*Cul4b*-null^ cells, and the loss of TiPARP in addition to the loss of CUL4B in MEF^*Cul4b*-null/*Tiparp*^ ^K.D.^ cells produced a greater accumulation of the AHR in the nucleus (TCDD *versus* C, +7.8-fold). Figure 4*C* shows that in the cytoplasmic fraction of TCDD-treated WT cells, the AHR was hardly detectable, whereas there was a faint band in the cytoplasmic fractions of TCDD-treated MEF^*Cul4b*-null^ and MEF^*Cul4b*-null/*Tiparp*^ ^K.D.^ cells (TCDD *versus* C, 80% decrease within each cell line). We then validated the findings obtained by Western blotting by immunofluorescence studies. Figure 4*D* (panels and bar graph) shows that TCDD treatment did not lead to AHR degradation in MEF^*Cul4b*-null/*Tiparp*^ ^K.D^ and that the AHR signal was detected in the nucleus (while it was detected mainly in the cytoplasm in solvent-treated cells), consistent with the Western blot results shown in Figures 2*C* and 4*C*, which show that the AHR is not degraded and accumulates in the nucleus after TCDD treatment in MEF^*Cul4b*-null/*Tiparp*^ ^K.D.^ cells. Inhibition of nuclear export by LMB treatment had no further effect on AHR levels after TCDD treatment in MEF^*Cul4b*-null/*Tiparp*^ ^K.D.^ cells, indicating that the loss of CUL4B and TiPARP is sufficient to inhibit the nuclear export of the AHR. In summary, these results show that in the absence of CUL4B and TiPARP, the AHR is not exported from the nucleus to the cytoplasm and accumulates in the nucleus.

### TiPARP is a major factor promoting AHR nuclear export and degradation after TCDD treatment

**Figure 5:**
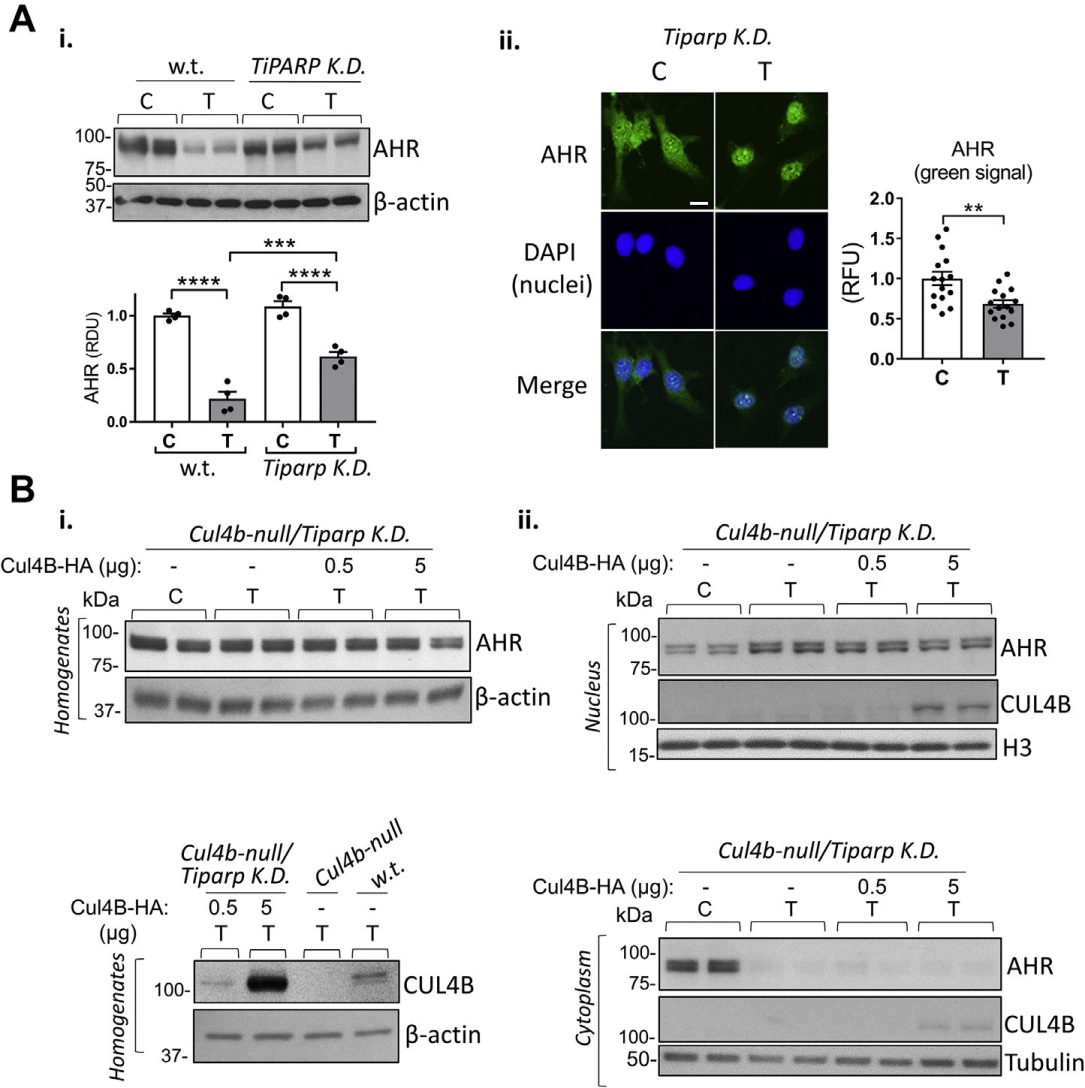
**TiPARP is a major factor promoting AHR nuclear export and degradation after TCDD treatment.***A*, i, WB using homogenates of WT MEF and MEF^*Tiparp*^^K.D.^ treated with the solvent (C) or TCDD (1 nM, 6 h). Antibodies against AHR and β-actin were used. The bar graph represents densitometry analysis of WB bands (means ± SEM). ii, representative images of immunofluorescence analysis in MEF^*Tiparp*^^K.D.^ treated with the solvent or TCDD (1 nM, 6 h) using an AHR antibody (FITC, *green*). DAPI, nuclear marker (*blue*). Merge, visualization of both AHR and nuclear signals. The bar graph represents the means ± SEM (n = 15 cells per treatment group) of AHR fluorescence signal (*green*). The *white* scale bar represents 20 μm. *B*, i, representative WB using homogenates of MEF^*Cul4b*-null*/Tiparp*^^K.D.^ transfected with CUL4B-HA construct (0.5 or 5 μg) and treated with the solvent or TCDD (1 nM, 6 h); antibodies against AHR and β-actin were used. *Lower panels*, WB using homogenates of WT MEF, MEF^*Cul4b*-null^, and MEF^*Cul4b*-null*/Tiparp*^^K.D.^ transfected with the CUL4B-HA construct (0.5 or 5 μg) and treated as above; antibodies against CUL4B and β-actin were used. ii, WB using nuclear- and cytoplasmic-enriched fractions of MEF^*Cul4b*-null*/Tiparp*^^K.D.^ transfected and treated as described in panel *B*i. Antibodies against AHR, CUL4B, and H3 were used. AHR, aryl hydrocarbon receptor; C, control (solvent); CUL4B, cullin 4B; DAPI, 4′,6-diamidino-2-phenylindole; MEF, mouse embryonic fibroblast; MEF^*Cul4b*-null^, MEF cell line in which the *Cullin 4B* gene had been knocked out; n.s., not significant; RDU, relative densitometry units; T, TCDD, T, 2,3,7,8-tetrachlorodibenzo-p-dioxin; TiPARP, TCDD-inducible poly(ADP-ribose) polymerase; WB, Western blotting.

To deepen the understanding of the role of TiPARP in regulating AHR nuclear export and degradation, we used WT MEF cells and CRISPR/Cas9 technology as described for Figure 2*C* to abolish TiPARP. Ultra-deep sequencing analysis showed that we obtained a 100% homozygous mutant cell line that harbors 99.8% of frameshift mutations leading to premature stop codons within the ORF of the TiPARP gene (Fig. S1). We treated MEF^*Tiparp*^ ^K.D.^ and WT MEF cells with solvent (control) or TCDD (1 nM, 6 h). Figure 5*A*i shows that TCDD-treated MEF^*Tiparp*^ ^K.D.^ cells had higher AHR levels (about 2-fold) than TCDD-treated WT cells. Furthermore, immunofluorescence studies (Fig. 4*A*ii) showed that AHR protein accumulated in the nucleus of TCDD-treated MEF^*Tiparp*^ ^K.D.^ cells. Similar AHR levels in TCDD-treated MEF^*Tiparp*^ ^K.D.^ cells were obtained by Western blotting and immunofluorescence (TCDD *versus* C, −30%; bar graphs, Fig. 5*A*i and ii). We also assessed AHR levels and localization in the absence of TiPARP by reintroducing CUL4B in MEF^*Cul4b*-null/*Tiparp*^ ^K.D.^ cells. MEF^*Cul4b*-null/*Tiparp*^ ^K.D.^ cells were transfected with a CUL4B construct (0.5 or 5 μg) and treated with the solvent or TCDD (1 nM, 6 h). Figure 5*B* shows that overexpressing CUL4B produced a minor decrease of AHR levels in both homogenates (*left panels*) and nuclear fractions (*right panels*) of MEF^*Cul4b*-null/*Tiparp*^ ^K.D.^ cells after TCDD treatment, even when overexpressed CUL4B (+5 μg CUL4B-HA) was about 80-fold higher than the levels of endogenous CUL4B in WT cells (Fig. 5*B*i lower panels, comparing bands in lanes 2 and 4). In summary, these results show that TiPARP has a greater effect than CUL4B in promoting TCDD-activated AHR nuclear export and proteasomal degradation.

## Discussion

AHR degradation, which occurs after TCDD activation, is an important event in regulating AHR signaling (10, 12, 13). Using genetically engineered MEF cell lines, we studied the roles of two factors that affect AHR protein stability: the E3 ubiquitin ligase CUL4B^AHR^ complex and the AHR target gene, TiPARP. We report here the discoveries that are as follows: (1) CUL4B and TiPARP collaborate to promote AHR protein degradation; (2) TiPARP has a greater effect than CUL4B in promoting AHR proteasomal degradation; and (3) loss of both CUL4B and TiPARP completely prevent TCDD-induced AHR degradation. We further showed that loss of CUL4B and TiPARP can have major effects on ligand-activated AHR transcriptional activity and action.

Export of the AHR from the nucleus to the cytoplasm is required for ligand-activated AHR proteasomal degradation (10). We showed here that loss of TiPARP caused accumulation of the TCDD-activated AHR in the nucleus, indicating that TiPARP participates in the regulation of ligand-activated AHR nuclear export. The AHR contains a nuclear localization signal in the N-terminal region and NESs both in the N-terminal region and in other domains (30, 31, 32, 33). Conformational changes of the activated AHR could reveal the NES leading to AHR nuclear export (34), and posttranslational modifications of the AHR could play a role in this process. TiPARP is a member of the PARP family, enzymes which target proteins for ADP-ribosylation, leading to different biological effects (25, 35), including inhibition or promotion of protein nuclear export (36, 37). Interestingly, Gomez *et al.* (38) recently reported, using *in vitro* studies, that TiPARP can mono-ADP-ribosylate the AHR at multiple sites (aa 430–848), including sites within the AHR Q-rich domain, which has been reported to contain a motif regulating the N-terminal NES and the AHR nuclear export to the cytoplasm (39, 40). It will be of interest to validate the role of the specific TiPARP-mediated ADP-ribosylation sites in promoting AHR nuclear export.

ADP-ribosylation by PARP enzymes has been reported to signal proteins for ubiquitination and proteasomal degradation (41, 42, 43) by the recruitment of E3 ubiquitin ligases to the ADP-ribosylated site of the proteins (25). Interestingly and relevant to our findings, a recent report (44) showed that TiPARP ADP-ribosylates and targets for proteasomal degradation HIF1A and other nuclear transcriptional factors, that is, c-MYC and the estrogen receptor, by forming nuclear complexes with the E3 ubiquitin ligase HUWE1 and several other E3 ubiquitin ligases. We showed here that AHR degradation still occurs *via* the 26S proteasome in the absence of the E3 ubiquitin ligase CUL4B as MG132 treatment abolished AHR degradation in TCDD-treated MEF^*Cul4b-null*^ cells (Fig. 1*D*), supporting the role for another E3 ubiquitin ligase besides CUL4B^AHR^ in AHR degradation. Further studies are needed to understand whether HUWE1 or another reported TiPARP-binding E3 ubiquitin ligase is involved in activated AHR proteasomal degradation.

We showed also that TiPARP and CUL4B have additive effects in curtailing AHR transcriptional activity as increased AHR protein levels caused by the loss of CUL4B or both CUL4B and TiPARP lead to enhanced TCDD induction of *Cyp1a1* and *1b1* mRNAs (Figs. 1*B* and 2, *B* and *D*). These findings are consistent with previous reports that pharmacological inhibition of AHR proteasomal degradation (10) and silencing TiPARP (20, 45) led to increased ligand-activated AHR levels and AHR transcriptional activity. It will be worthwhile in the future to study whether SNPs that affect the function of TiPARP (and CUL4B) can exacerbate or protect against the effects of TCDD and other environmental toxins that activate the AHR. *In vitro* studies using human TiPARP constructs harboring identified human SNPs showed that TiPARP SNPs can affect AHR activation (Ahmed 2015). No known human CUL4B SNPs have been identified so far.

We also present the unexpected finding that loss of CUL4B abrogated the induction of *Infb1* mRNA both in control and TCDD-treated cells (Fig. 3), suggesting that CUL4B might affect *Infb1* mRNA induction by 3pRNA independently of AHR activation. These interesting findings will require further studies.

Questions regarding AHR ubiquitination, nuclear export, and consequent degradation remain. For example, Is TiPARP-mediated ADP-ribosylation of the AHR required to signal for ubiquitination as has been reported for other proteins that undergo ubiquitination after being ADP-ribosylated by PARP enzymes? (25, 44). Where does AHR ubiquitination occur? The evidence that CUL4B has been reported to be predominantly localized in the nucleus (15, 21) and that Ohtake *et al.* (16) identified and characterized the CUL4B^AHR^ protein complex from nuclear extracts support the hypothesis that the AHR is ubiquitinated before being exported from the nucleus to the cytoplasm.

Furthermore, although there is some evidence that the AHR can be degraded in the nucleus in the absence of a ligand (24), other reports from Pollenz (12, 13) and our findings (Fig. 4*A*) showing that the nuclear export inhibitor LMB prevents TCDD-activated AHR degradation support that the AHR degradation occurs by the 26S proteasome in the cytoplasm after TCDD-activated AHR nuclear export.

In summary, we provided evidence that the CUL4B^AHR^ complex and TiPARP have important roles in AHR protein degradation and action, showing that (1) CUL4B^AHR^ and TiPARP have collaborative effects on AHR protein levels, (2) CUL4B^AHR^ has a lesser effect than TiPARP on AHR degradation, and (3) TiPARP is a major factor needed for TCDD-activated AHR translocation from the nucleus to the cytoplasm and consequent proteasomal degradation.

## Experimental procedures

### Cells, reagents, and constructs

MEF^*Cul4b-null*^ cell line was originally derived from the CUL4B KO mouse described in ref (21) in the laboratory of P.Z. The WT MEF cell line was also derived from the control mouse used in those studies (21). Cell lines were maintained in Dulbecco's modified Eagle's medium (DMEM) supplemented with 10% fetal bovine serum (FBS) (ATCC) and 1% Penicillin-Streptomycin solution (Invitrogen). Cells were cultured at least for 24 h before the addition of treatment compounds directly in the medium (without medium change) to avoid possible activation of the AHR in control samples (46). Other reagents and their sources were as follows: TCDD (MRIGlobal Chemical Carcinogen Repository); MG132, 10 mM ready-made solution in DMSO from Sigma; LMB, 25 μg/ml solution in ethanol from Abcam; mouse HA-CUL4B vector was provided by P. Z.

### RNA extraction and real-time quantitative PCR

MEF cells were seeded at 0.3 × 10^6^ cells/well in 6-well plates (Corning Inc) containing DMEM supplemented with 10% FBS and 1% Penicillin-Streptomycin solution. The next day, cells were treated for 6 h with TCDD or dioxane at the concentration indicated in each figure. RNA STAT-60 (Tel-Test “B”) was used for total RNA extraction, following the manufacturer’s directions. For preparation of cDNA, 0.8 to 1 μg of total RNA was mixed with 4 μl of qScript cDNA SuperMix (Quanta Biosciences) and nuclease-free water to 20-μl total reaction volume. The mixture was incubated sequentially at 25 °C for 5 min, at 42 °C for 40 min, and 85 °C for 5 min. The cDNA obtained was diluted in water 1:5. Real-time quantitative PCR (RT-qPCR) was carried out in a 20-μl reaction mixture containing 2 μl of cDNA, 10 μl PerfeCTa SYBR Green FastMix (Quanta Biosciences), 1 μl forward and reverse primers (10 μM), and the remaining volume of nuclease-free water. The primers used for qPCR amplification and their corresponding annealing temperatures are shown in the Table S1.

### siRNA-mediated gene silencing

WT MEF or MEF^*Cul4b*-null^ cells were seeded at cell densities of 0.5 × 10^6^ cells/well in 6-well plates. Cells were transfected the following day with 1.25-μg siGENOME Mouse TiPARP siRNA (M-060174-01-0005, Dharmacon) or 1.25-μg siGENOME Non-Targeting siRNA Pool #2 (D-001206-14-05, Dharmacon) in 7.5-μl Lipofectamine 3000 (Invitrogen) following the manufacturer’s instructions. Twenty-four hours after transfection, cells were treated with 10 nM TCDD or the solvent dioxane (control). Six hours after treatment, cells were scraped in 1-ml RNA STAT-60 for RNA extraction and RT-qPCR analysis or in 300-μl 2× sample buffer for Western blotting analysis.

### Preparation of cell lysates for SDS-PAGE/Western blotting

MEF cells were scraped in 300-μl 2× sample buffer (125 mM Tris HCl, 4% SDS, 16% glycerol, 10% β-mercaptoethanol, 0.002% bromophenol blue) and boiled for 5 min. Total protein concentrations were measured by Bio-Rad assay following the manufacturer’s instructions. For Western blotting, equal amounts of protein from each sample, up to 30 μg per lane, were separated on precast Tris-Glycine gels (Invitrogen) and transferred to nitrocellulose membranes. Primary antibodies and their dilutions were as follows: anti-AHR (BML-SA210; Enzo Life Sciences), 1:1000; anti-ARNT (sc-17811, Santa Cruz Biotechnology), 1:200; anti-CUL4B (12916-1-AP; ProteinTech), 1:1000; anti-β actin (A5441, Sigma) 1:50,000; anti-histone-3, H3 (H0164, Sigma), 1:30,000, and anti-α/β tubulin (2168, Cell signaling), 1:1000. Secondary antibodies were peroxidase-conjugated goat anti-rabbit (A6154, Sigma) or mouse IgG kappa binding protein (sc-516102S, Santa Cruz Biotechnology). Protein bands were detected with ECL Western Blotting Detection reagents (GE Healthcare). Band intensities were measured by densitometry and were normalized to β-actin, α/β tubulin, or histone 3 (H3) levels using GeneTools analysis software (Syngene).

### CRISPR/Cas9–mediated suppression of TiPARP in WT MEF and MEF^Cul4b-null^ cell lines

WT MEF or MEF^*Cul4b*-null^ cells were resuspended in Mouse ES Cell Nucleofector Solution (Lonza Inc) at 1 × 10^6^ cells/0.1 ml. Each 0.1-ml cell suspension was mixed with ribonucleoprotein particles consisting of 1.2 μmol of synthetic sgRNA + 150 pmol of Cas9 2NLS protein (Synthego) and 2 μg GFP plasmid (Lonza Walkersville, Inc) to control transfection efficiency. For TiPARP gene KO, the following sgRNA sequence recognizing the nucleotides from position 973 to 989 in the exon 2 of the mouse *TiPARP* gene was used: CTGCAAGGCACTTGCATTTA (CRISPRevolution sgRNA EZ Kit, modified; Synthego). Transfections were performed using program A023 with Amaxa Nucleofector device following the manufacturer's instructions. Cells were plated, and after 48 h, they were collected and re-seeded with serial dilutions in 96-well culture plates to achieve a cell population with enriched mutations. Cell populations were then analyzed for TiPARP mutations by AMPLICON-EZ next-generation sequencing at GENEWIZ Global Headquarters using the following primers: Forward, 5’-GCTTCCCTTGAGCTTGTGTT-3’; Reverse, 5’- TGGAAACACTCTGCCACTTCT-3’.

### Cell treatment and transfection with viral motifs for analysis of Infb1 gene expression

WT MEF, MEF^*Cul4b*-null^, and MEF^*Cul4b*-null/*Tiparp*^ ^K.D.^ cells were seeded at cell densities of 0.3 × 10^6^ cells/well in 6-well plates. The following day, cells were treated with 1 nM TCDD or dioxane. After 1 h, cells were transfected with 1 μg/ml of triphosphate dsRNA (3pRNA) or its negative control (3pRNAc) (InvivoGen) in 7.5-μl Lipofectamine 3000, following the manufacturer’s instructions. Twenty-four hours after transfection with 3pRNA, cells were scraped in 1-ml RNA STAT-60 for RNA extraction and RT-qPCR analysis to assess *Infb1* mRNA expression using the primers shown in the Table S1.

### Subcellular nuclear and cytoplasmic fraction preparation

Cytoplasmic- and nuclear-enriched fractions from MEF cells were obtained by using the NE-PER Nuclear and Cytoplasmic Extraction kit (78833; Invitrogen) following the manufacturer’s instructions with the following modifications: CER II buffer was added at a ratio of 0.68 μl per 10 μl of packed cell pellet, and vortexing was avoided at all times. After addition of buffer CER II, lysed cells were centrifuged for 10 min (1000*g*). The supernatant (cytoplasmic fraction) was snap-frozen, while the pellet (nuclear fraction) was washed once with 1× PBS before being snap-frozen. The subcellular fractions were used for Western blotting analysis following the same procedures indicated above. To assess the enrichment of the nuclei and cytoplasmic fractions, primary antibodies anti-histone-3 H3, nuclear marker, and anti-α/β tubulin, cytoplasmic marker, were used.

### Immunofluorescence analysis

To visualize AHR protein in MEF cells by immunofluorescence, cells were seeded in 6-well plates on poly-D-lysine–coated glass coverslips at a cell density of 0.12 × 10^6^ cells/well in DMEM supplemented with 10% dialyzed FBS (Invitrogen) and 1% Penicillin-Streptomycin solution (Invitrogen). The next day, cells were pretreated with LMB (50 nM, 16 h) or solvent ethanol, and followed by TCDD treatment (10 nM, 6 h) or solvent dioxane (control). After treatments, cells were fixed in a 1:1 mixture of cold MetOH:acetone at −20 °C for 10 min. Cells were then air-dried and blocked in 10% normal goat serum (50197Z, Invitrogen) + 1% BSA in 1× PBS for 1 h at room temperature (RT). Cells were then incubated with an AHR primary antibody (BML-SA210, Enzo Life Sciences; 1:100) overnight at 4 °C. For detection, cells were rinsed with 1× PBS two times and then incubated with a secondary antibody goat anti-rabbit conjugated with Alexa Fluor 488 green fluorescent dye (A-11008, Invitrogen; 1:500) for 1 h at RT. Glass coverslips were then rinsed two times with 1× PBS and mounted on microscope slides using an anti-fade solution (S36967, Invitrogen) and examined using a fluorescence microscope (Nikon Eclipse TE2000-E). 4′,6-Diamidino-2-phenylindole solution (62248, Invitrogen) was used as the nuclear counterstain. Fluorescence intensity measurements of AHR green signal were obtained using ImageJ software (Rasband, W.S., ImageJ, U. S. National Institutes of Health, Bethesda, MD, http://imagej.nih.gov/ij/, 1997–2016).

### Statistics

Statistical significance of the differences between group means was evaluated by one-way ANOVA using Tukey's honestly significant difference test as a post hoc test; *p* values ≤0.05 were considered statistically significant.

## Data availability

All data are contained in the article and in the Supporting information.

## Supporting information

This article contains supporting information.

## Conflict of interest

The authors declare that they have no conflicts of interest with the contents of this article.
