# Supplementary material for: Roles of the ubiquitin ligase CUL4B and ADP-ribosyltransferase TiPARP in TCDD-induced nuclear export and proteasomal degradation of the transcription factor AHR
Source: J Biol Chem. 2021 Jun 16;297(2):100886. doi: 10.1016/j.jbc.2021.100886 (PMC8318916; doi:10.1016/j.jbc.2021.100886)
Supplement: Supplemental Figure S1 and Table S1 [file mmc1.pdf]

SUPPORTING INFORMATION

Table S1. Primer sequences and annealing temperatures (Tm) used for quantification of mRNAs by real-time qPCR.

**Target**	**Primer sequence**	**Tm**
18S	**F:** 5′- CGCAGCTAGGAATAATGGAA-3′	54 °C
	**R:** 5′- TCTGATCGTCTTCGAACCTC-3′	
*Cyp*1a1	**F:** 5′-TGCCTTCCATGTATGGACTT-3′	53 °C
	**R:** 5′-GTCAGCATGTGACCAATGAA-3′	
*Cyp*1b1	**F:** 5′-AACCCAGAGGACTTTGATCC-3′	54 °C
	**R:** 5′-ATGGTCAGGCCATAACTGAA-3′	
Infb1	**F:** 5′-GAGCTCCAAGAAAGGACGAAC-3′	58 °C
	**R:** 5′-GGCAGTGTAACTCTTCTGCAT-3′	
Tiparp	**F:** 5′-ACTTTGATCCCCGTGTCTGT-3′	55 °C
	**R:** 5′-GCCCATTGTGTATCTGCCAG-3′	

Supplementary Figure 1 - A, Summary of results obtained by ultra-deep sequencing of PCR products using next generation sequencing (Amplicon-EZ NGS, Genewiz) amplifying TiPARP exon 2 region targeted by CRISPR/CAS9 in CUL4B/TiPARP DKO or TiPARP KO MEF cells. **B**, Most abundant frameshift mutations identified with next generation sequencing as described in A**.** In red, sequence recognized by the single-guided RNA (sgRNA). In blue, base insertions; '-', base deletions.

A.

Sample	TargetReads[1]	MutantReads[2]	MutantPct(%)	Genotype	FrameshiftMutantReads[3]	FrameshiftMutantPct(%)
**CUL4B/TiPARP DKO MEF**	150642	149436	99.2	Homozygous Mutant	145410	96.53
**TiPARP KO MEF**	68763	68667	99.86	Homozygous Mutant	68636	99.82

[1]Target Reads: Number of PCR product reads in target region.
[2]Mutant Reads: Number of PCR product reads with base insertion/s and/or deletion/s.
[3]Frameshift Mutant Reads: Number of PCR product reads harboring frame shift mutations.

Read: PCR product with a unique sequence.

B.

Sample	Frameshift Mutation Sequences	%	Type
CUL4B/TiPARP DKO MEF	CTTTCTGCAAGGCACTTGCATttTTATGGCAGGGATT	33.42	Insertion
	CTTTCTGCAAGGCACTTGCATgtTTATGGCAGGGATT	33.09	Insertion
	CTTTCTGCAAGGCACTTGCATtTTATGGCAGGGATT	11	Insertion
	CTTTCTGCAAGGCACTTGCA-TTATGGCAGGGATT	5.25	Deletion
	CTTTCTGCAAGGCACTTGCA--TATGGCAGGGATT	4.65	Deletion
	CTTTCTGCAAGGCACTTGCAtaTTTATGGCAGGGATT	0.95	Insertion
TiPARP KO MEF	CTTTCTGCAAGGCACTTGCATtTTATGGCAGGGATT	50.86	Insertion
	CTTTCTGCAAGGCACTTGC--TTATGGCAGGGATT	17.05	Deletion
	CTTTCTGCAAGGCACTTGC-TTTATGGCAGGGATT	15.38	Deletion
	CTTTCTGCAAGGCAC-----TTTATGGCAGGGATT	12.64	Deletion
	CTTTCTGCAAGGCACTTGCAaTTTATGGCAGGGATT	0.39	Insertion
	CTTTCTGCAAGGCACTTGCAtaTTTATGGCAGGGATT	0.22	Insertion
